# Successful therapy using high-dose furmonertinib for non-small cell lung cancer with leptomeningeal metastasis: a case report and literature review

**DOI:** 10.3389/fonc.2023.1233198

**Published:** 2023-10-18

**Authors:** Ting Chen, Jie Chen, De-sheng Liu, Yan-ling Shu, Mao-yue Fu, Hai-jun Gou, Kai-jian Lei, Yu-ming Jia

**Affiliations:** ^1^ Department of Oncology, Second People’s Hospital of Yibin, Yibin, Sichuan, China; ^2^ Department of Thoracic and Cardiovascular Surgery, Second People’s Hospital of Yibin, Yibin, Sichuan, China; ^3^ Department of Oncology, People’s Hospital of Junlian County, Yibin, Sichuan, China

**Keywords:** NSCLC, *EGFR*, furmonertinib, leptomeningeal metastasis, target therapy

## Abstract

**Background:**

Lung cancer is the second most common form of malignant tumor and has the highest mortality rate worldwide. Among its subtypes, lung adenocarcinoma is the most prevalent. Leptomeningeal metastasis (LM) is rare and is characterized by a dismal prognosis, with overall survival periods typically spanning 4 to 6 weeks without treatment. However, in specific cases, survival can be extended to 4 to 6 months with appropriate therapy. The recent approval of third-generation tyrosine kinase inhibitors (TKIs), such as osimertinib, aumolertinib, and furmonertinib, has introduced promising treatment options for individuals with non-small cell lung cancer (NSCLC) who develop LM after developing resistance to first- and second-generation TKIs. These third-generation TKIs exhibit an enhanced ability to penetrate the blood–brain barrier (BBB), opening up new avenues for managing this challenging condition.

**Case summary:**

We report the case of a 48-year-old Chinese man diagnosed with advanced NSCLC harboring an epidermal growth factor receptor (*EGFR*) mutation. Following a pulmonary lobectomy and postoperative adjuvant therapy with gefitinib, the patient was diagnosed with LM, which was confirmed by his neurologic symptoms, cerebrospinal fluid cytologic analysis, and cranial enhancement magnetic resonance imaging. Subsequently, he received oral treatment in the form of 160 mg of furmonertinib daily. After 5 days of furmonertinib therapy, the patient recovered from lethargy, with an obvious improvement in cognitive function. Follow-up visits revealed a 6-month survival period following the LM diagnosis. Patients with NSCLC and LM typically present with severe symptoms, and the efficacy of systemic treatment, intrathecal chemotherapy, and radiotherapy remains unsatisfactory. We hope that this specific case provide valuable insights into the management of patients with *EGFR* mutation-associated NSCLC with LM.

**Conclusion:**

Furmonertinib, a third-generation *EGFR* TKI with notable BBB penetration, shows promise in LM control and the rapid alleviation of intracranial symptoms. Further investigations into appropriate dosage and toxicity management are imperative.

## Introduction

Lung cancer is the second most common form of malignant tumor worldwide, and it also has the highest mortality rate worldwide (1). Among the various subtypes, adenocarcinoma emerges as the most prevalent. Notably, gene mutations within the epidermal growth factor receptor (*EGFR*) represent the most prevalent driver mutations, with *EGFR*-activating mutations being detected in approximately 10% of Caucasians and 30% to 40% of East Asians (2). For patients with advanced non-small cell lung cancer (NSCLC) and *EGFR* mutations, first- and second-generation *EGFR* tyrosine kinase inhibitors (TKIs) have been approved as the first-line treatment. However, acquired resistance is inevitable in most cases, as a result of the *EGFR* T790M mutation, among patients who benefit from these *EGFR* TKIs. The central nervous system (CNS) is a common site of metastasis, including brain metastasis (BM) and leptomeningeal metastasis (LM), partly because of the limited blood–brain barrier (BBB) penetration of the first- and second-generation TKIs (3). The incidence of LM is 3.8% among all patients and can increase to 10% in those with NSCLC and *EGFR* mutations (4). Prior to the advent of TKIs and immunotherapy, the treatment of LM showed limited improvement in overall survival (OS); this treatment involved intrathecal chemotherapy (ITC), whole-brain radiation therapy (WBRT), and systemic chemotherapy. Although LM is relatively rare and bears a poor prognosis, with OS of only 4–6 weeks without therapy, a subset of patients can experience an extension in survival to 4–6 months with treatment (5). Recent years have seen the approval of third-generation TKIs, such as osimertinib, aumolertinib, and furmonertinib, targeting both *EGFR*-activating mutations and the T790M mutation, and with improved ability to diffuse through the BBB. These agents have offered promising treatment options for NSCLC with LM. However, despite the growing interest in CNS metastases in the modern era, most controlled clinical trials have excluded patients with LM due to their grim prognosis and poorer performance status. In addition, although a recent increase in incidence has occurred, LM is a rare complication of NSCLC and patient numbers remain limited, posing significant challenges for the implementation of random clinical trials. Moreover, previous studies have demonstrated significant genomic divergence between the primary tumor and intracranial metastases (6). Consequently, providing individualized therapy for patients with LM remains an exceptionally challenging task for clinical physicians.

Furmonertinib, developed in China, is a newly designed third-generation *EGFR* TKI with a trifluoroethoxypyridine-based molecule structure. The efficacy of furmonertinib in treating NSCLC with LM remains unclear. Herein we present the case of a patient with NSCLC who developed LM and underwent high-dose furmonertinib treatment. We aim to provide insights and valuable experience for the management of NSCLC patients with LM.

## Case presentation

**Figure 1:**
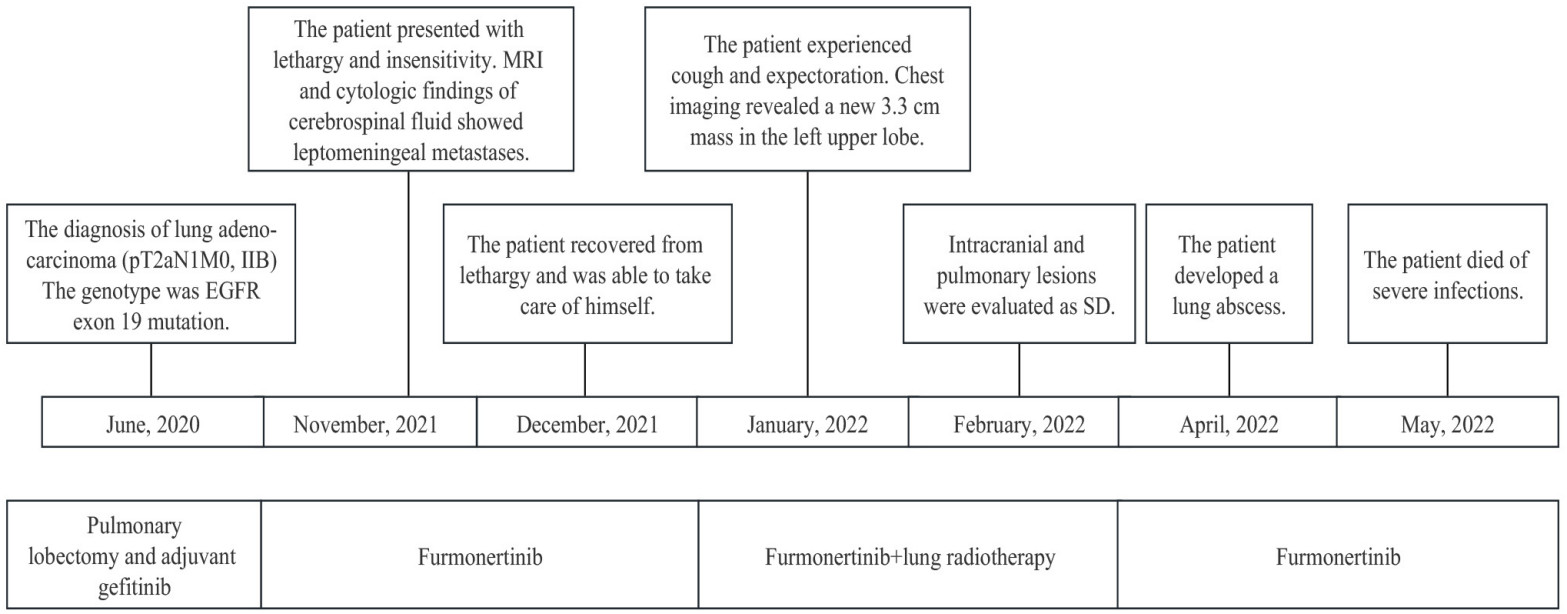
Summary of the diagnosis and treatment process.

**Figure 2:**
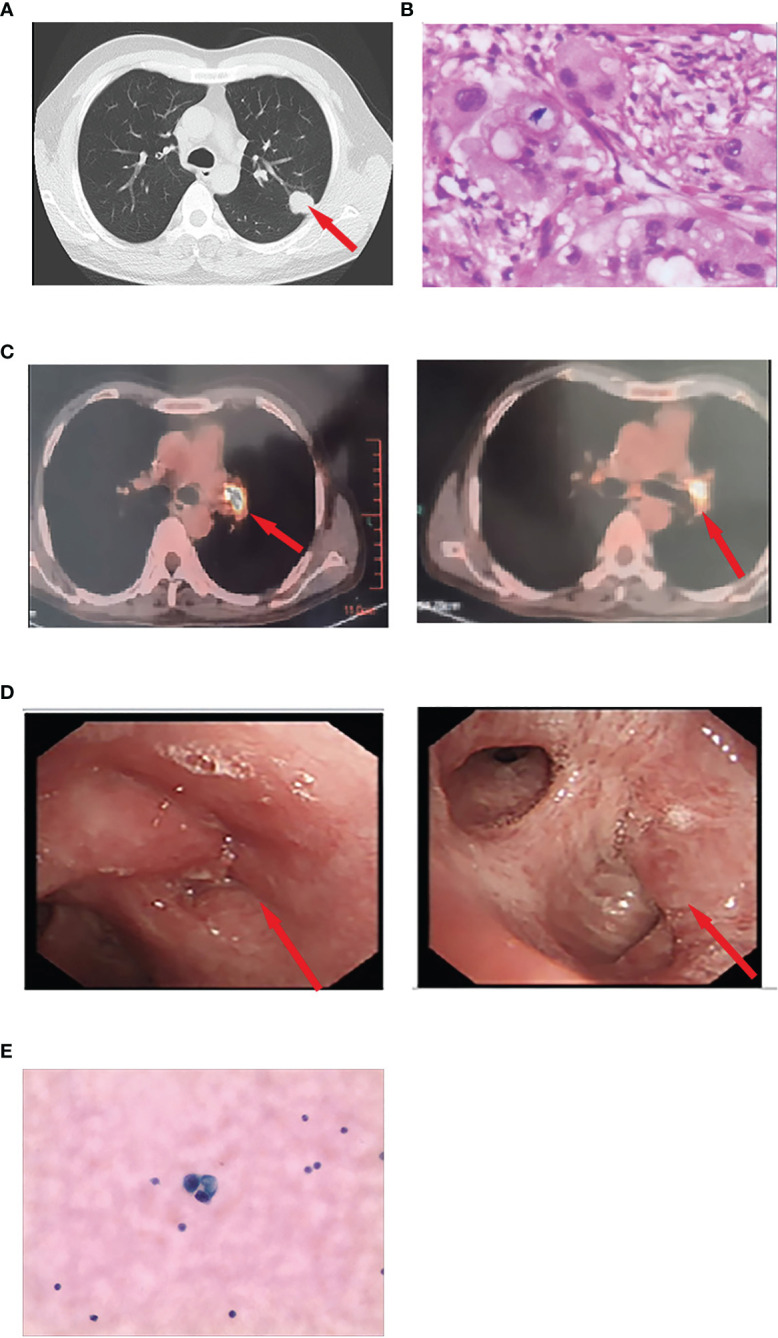
**(A)** Preoperative chest computed tomography revealing a 25 × 20-mm mass in the left upper lobe. **(B)** Pathological diagnosis of the resected specimen was lung adenocarcinoma. **(C)** Positron emission/CT examination showed abnormally increased fluorodeoxyglucose metabolism in the left hilar surgical region and mediastinal lymph nodes. The cytologic findings of CSF by lumbar puncture revealed adenocarcinoma cells. **(D)** An electronic fiber bronchoscopy revealed complete obstruction of the left upper lobe bronchus with a neoplasm protruding from the lumen at the opening and leading to left inferior basal segment external pressure stenosis. **(E)** The cytologic findings of CSF by lumbar puncture revealed adenocarcinoma cells.

A 48-year-old Chinese man with a long-term history of smoking was diagnosed with stage IIB lung cancer in June 2020 (**Figure 1**). Preoperative chest computed tomography (CT) revealed the presence of a 25 mm × 20 mm mass in the upper lobe of the left lung, deemed resectable upon surgical evaluation (**Figure 2A**). Thereafter, he underwent thoracoscopic-assisted pulmonary radical lobectomy and lymph node dissection. Pathologic examination identified adenocarcinoma with positive staining for Napsin A, TTF-1, p53, and CK7 and negative staining for p63, CK5/6, and Ki-67 (30%). Parabronchial lymph nodes (2/4) showed negative staining for CK5/6 and P63. Pleural invasion and vascular cancer embolism were observed (pT2aN1M0 stage IIB; AJCC 8th edition) (**Figure 2B**). The patient declined postoperative chemotherapy and radiotherapy. The surgical specimen was analyzed using next-generation sequencing (NGS) to identify possible targetable molecular alterations. The findings revealed a mutation in exon 19 of *EGFR*, with no other gene mutations detected.

**Figure 3:**
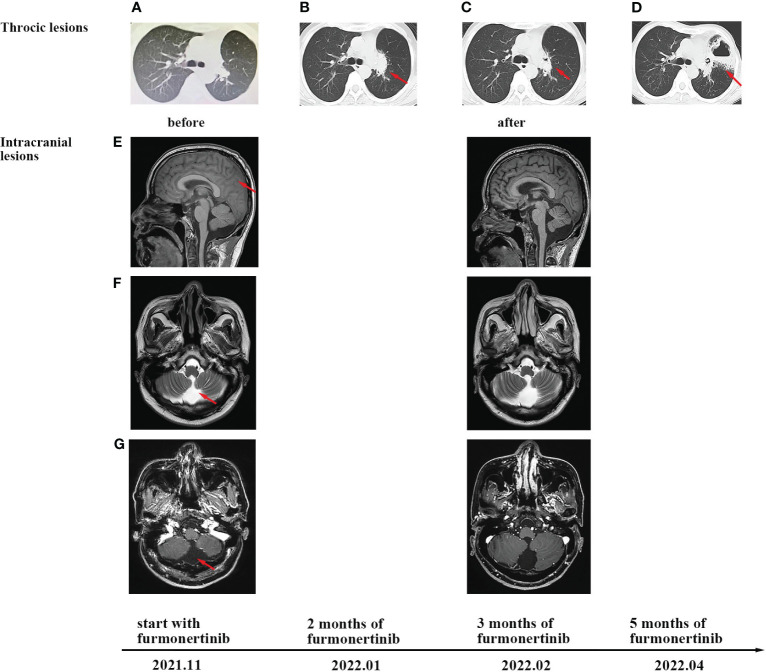
CT and MRI scans at different clinical time points, as indicated. **(A)** Chest CT scans before furmonertinib treatment. **(B)** Progression of thoracic lesions was observed after 2 months of furmonertinib. **(C)** The change in lung lesions after 3 months of furmonertinib and lung radiotherapy. **(D)** CT scans after 5 months of furmonertinib revealed a left-lung abscess. **(E)** T1-weighted imaging of brain MRI before and after 3 months of furmonertinib. The coronal plane revealed slightly swollen meninges. **(F)** T2-weighted imaging of brain MRI indicated mild hydrocephalus. **(G)** T1-weighted imaging enhancement scans revealed no leptomeningeal enhancement and no parenchyma metastases.

Subsequently, the patient received adjuvant targeted therapy with the first-generation TKI gefitinib (250 mg orally) every day for 15 months, during which time no recurrence was noted. However, due to transaminase elevation (grade 2, common terminology criteria for adverse events), dyspepsia (grade 2), and rash (grade 2), the patient temporarily withdrew without medical permission. In November 2021, the patient experienced rapid disease progression, with an Eastern Cooperative Oncology Group (ECOG) performance status (PS) of 3. He exhibited a series of symptoms, including nausea, vomiting, dizziness, lethargy, insensitivity, and an inability to provide accurate answers to questions. Brain enhancement magnetic resonance imaging (MRI) examination revealed mild hydrocephalus and slightly swollen meninges without parenchyma metastases (**Figure 3**). No extracranial recurrence was detected following a comprehensive examination. A second round of NGS using peripheral blood confirmed an *EGFR* exon 19 mutation, with no evidence of acquired T790M, *KRAS*, *ALK*, *ROS1*, *RET*, or *MET* mutations. Cytologic findings from cerebrospinal fluid (CSF) obtained by lumbar puncture revealed adenocarcinoma cells (**Figure 2E**). Furmonertinib is a novel third-generation *EGFR* TKI that targets both *EGFR*-sensitive mutations and the T790M mutation while sparing wild-type *EGFR*. Furmonertinib has been shown to be more effective than gefitinib in treating the CNS in patients with *EGFR*-mutated NSCLC and CNS metastases. Therefore, our patient was administered high-dose furmonertinib (160 mg/day). Mannitol was administered to alleviate cerebral edema. Remarkably, within 5 days of receiving furmonertinib, the patient, who had been admitted with lethargy, showed significant improvement in physical and cognitive function. After discharge, the patient continued oral furmonertinib at a daily dose of 160 mg. Imaging studies were not performed until January 2022, following 2 months of furmonertinib administration, when the patient developed symptoms of cough, expectoration, and dyspnea. A chest examination revealed a new 3.3 cm mass in the left upper lobe hilar region, with right hilar and mediastinal lymphadenopathies (**Figure 3**). Electronic fiber bronchoscopy revealed complete obstruction of the left upper lobe bronchus, with a neoplasm protruding from the lumen at the opening (**Figure 2D**). A biopsy was attempted but proved to be challenging, yielding inconclusive pathological results. Re-biopsy was quite difficult to perform. Positron emission tomography/CT (PET/CT) indicated abnormally increased fluorodeoxyglucose metabolism in the left hilar surgical region, left thoracic entrance, mediastinal aortic arch, right lower paratracheal lymph nodes, carina lymph nodes, and right hilar lymph nodes (**Figure 2C**). While definitive pathological diagnoses were unavailable, a combination of CT, PET/CT, and bronchoscopy findings led us to suspect tumor recurrence in the lungs. Consequently, the patient received intensity-modulated radiotherapy at a total dose of 30 Gy, delivered over a 2-week period at 3 Gy per fraction, in conjunction with 160 mg (daily) of furmonertinib. By February 2022, after 3 months of furmonertinib and radiotherapy, both intracranial and pulmonary lesions were evaluated as stable disease (SD) (**Figure 3**). The primary adverse events observed were transaminase elevation (grade 2) and nausea (grade 2). In April 2022, following 5 months of furmonertinib administration, the patient was hospitalized due to a left lung abscess, accompanied by fever, cough, dyspnea, and loss of consciousness. Anti-infective therapy was administered for 2 weeks. One month later, in May 2022, the patient died of severe infections. Accordingly, furmonertinib extended the patient’s OS by 6 months.

## Discussion

LM is primarily diagnosed using cerebrospinal fluid cytology, and brain MRI also exhibits special imaging features in which LM primarily or solely manifests as cranial nerve involvement (7). NSCLC with LM generally presents with serious symptoms, with headaches, nausea, and vomiting being the most common symptoms. LM carries a grim prognosis, often shorter than that of BM. The effectiveness of systemic treatment, ITC, and radiotherapy has been unsatisfactory. Systemic chemotherapy has a clear curative effect in the treatment of NSCLC patients with LM, as it is an independent predictor of survival (5). In the case of ITC, it shows a specific efficacy for NSCLC patients. A pooled analysis, comprising four prospective studies and five retrospective studies, evaluated 552 patients who received multiple interventions (ITC, WBRT, *EGFR* TKI, systemic chemotherapy, and supportive care) and 37 patients who received ITC only. The analysis reported a longer median OS among patients who received ITC only (6.0 months) compared to those who received multiple interventions (3.0–5.0 months) (8). However, given the heterogeneity in the ITC treatment and confounding factors, further research is needed to determine the efficacy of ITC. WBRT, on the other hand, has limited efficacy for NSCLC patients with LM. A retrospective study analyzed 51 NSCLC patients with LM and found no significant differences in intracranial objective response rate (ORR) (15.4% vs.16%, *p* = 0.952) or disease control rate (34.7% vs. 28%, *p* =0.611) between the WBRT group and the non-WBRT group. Survival was also not statistically improved for patients treated with WBRT (9). Another study reached the same conclusion, namely, that receiving WBRT did not confer a survival advantage (10). In clinical practice, WBRT is typically applied for palliative relief, for example to address obstructive lesions causing hydrocephalus (11). In a Phase II clinical trial in which WBRT was combined with ITC for solid tumors in patients with LM, the median OS was only 6.7 months among NSCLC patients (12). Moreover, a significant number of patients (those with a weak physical status) may not tolerate conventional chemotherapy and radiotherapy, necessitating individualized therapeutic strategies for LM patients. Previous studies have reported that a good ECOG PS is a significant prognostic factor. In one study, among patients diagnosed with LM who underwent ITC with or without systemic treatment, including cytotoxic chemotherapy and *EGFR* TKIs, the median OS was only 0.7 months in patients with an ECOG PS of 3–4, significantly shorter than the 5.5 months observed in patients with an ECOG PS of 1–2 (*p* < 0.001) (13). Poorer PS significantly impacts the efficacy of therapeutic regimens and limits treatment options for patients.

The National Comprehensive Cancer Network Guidelines recommend the use of osimertinib (regardless of T790M status) for patients with *EGFR*-activating mutations who have progressive CNS disease or leptomeningeal disease, based on the BLOOM clinical trial data (14). We have summarized recent clinical trials of third-generation TKIs on NSCLC patients with LM (**Table 1**) (15–22). In the previous AURA program studies (AURA extension, AURA2, AURA17, and AURA3), 22 patients with LM with acquired T790M resistance mutation received 80 mg of osimertinib after disease progression on prior TKIs. A retrospective analysis of this dataset showed a LM ORR of 55%, with median progression-free survival (PFS) of 11.1 months and OS of 18.8 months (17). Consequently, in the Phase I BLOOM study, 41 patients with cytologically confirmed LM received 160 mg of osimertinib. The LM ORR was 62%, with PFS of 8.6 months and OS of 11.0 months (19). Compared with 80mg osimertinib, 160 mg osimertinib slightly improved the LM ORR. The findings initially suggest that osimertinib may have promising effects on LM disease control, but further prospective clinical trials are needed to confirm its efficacy in LM and determine the optimal dose. Furmonertinib is a newly designed third-generation *EGFR* TKI with a trifluoroethoxypyridine-based molecule structure, demonstrating promising efficacy in patients with NSCLC having *EGFR* activation or T790M mutation. Preclinical studies have shown higher concentrations of drug-related active substances in the brain than in the plasma, indicating that furmonertinib may be effective in patients with CNS metastases (23). In the phase III FURLONG study, which enrolled 63 *EGFR*-sensitizing mutation-positive, untreated patients with asymptomatic CNS metastases, furmonertinib was associated with longer PFS [18.0 vs. 12.4 months, hazard ratio (HR) 0.50, *p*=0.0028], CNS PFS (20.8 vs. 9.8 months, HR 0.40, *p*=0.0011), and CNS ORR (91% vs. 65%, OR=6.82, *p*=0.0277) compared with gefitinib (24). In a Phase II dose-expansion study enrolling patients with NSCLC harboring the *EGFR* T790M mutation, the CNS efficacy of furmonertinib as a second- or later-line treatment was tested; the results showed promising CNS ORR (60.0% with 80 mg once daily and 84.6% with 160 mg once daily) and CNS PFS (9.7 months with 80 mg once daily and 19.3 months with 160 mg once daily) (25), indicating that a higher dose of furmonertinib may lead to better outcomes in patients with NSCLC having CNS metastases. However, the efficacy of double-dose furmonertinib needs further validation with balancing of baseline characteristics. Prior brain radiotherapy may affect the subsequent response to targeted drugs. In the radiotherapy subgroup analysis of the AURA3 study, the CNS ORR in patients with prior CNS radiotherapy-treated with osimertinib was 64%, which was higher than the 34% observed in radiotherapy-naive patients. Compared with the AURA3 study, the AST2818 study included more patients with *EGFR* L858R mutations (47.8% vs. 38.8%) and fewer patients with previous CNS radiotherapy (13% vs. 28.8%) (26, 27). Based on the data, the efficacy of furmonertinib in reducing intracranial lesions seems not to be inferior to that of osimertinib. Furthermore, previous cases have indicated that a high dose of furmonertinib could reverse osimertinib resistance in patients with NSCLC having BM (28, 29). In our case, the LM PFS was 6 months, and disease control of LM was observed even when the disease progressed in extracranial lesions, indicating the potential of furmonertinib for sustained efficacy in LM.

**Table 1:** Selected studies of third-generation TKIs in NSCLC patients with LM.

Study	Year	Type	Treatment	Patients (*n*)	*EGFR* status	Rate of T790M positivity	Median PFS (m)	Median OS (m)	LM ORR	LM DCR	Toxicity
Saboundji et al. (15)	2018	R	Osimertinib	20	2 Exon18 7 Exon19 11 Exon21	65%	17.2	18.0	85%	NA	NA
Nanjo et al. (16)	2017	P	Osimertinib 80mg/day	13	10 Eon19 3 Exon21	100%	7.2	NR	15%	NA	G3–4: 0%
Ahn et al. (17)	2020	R	Osimertinib 80mg/day	22	3 Exon18 13 Exon19 8 Exon21	100%	11.1	18.8	55%	91%	G3–4: 45%
Park et al. (18)	2020	II	Osimertinib 160mg/day	40	1 Exon18 23 Exon19 16 Exon21	100%	8.0	13.3	12.5%	92.5%	G3–4: 34%
Yang et al. (19)	2020	I	Osimertinib 160mg/day	41	EGFR mutation	100%	8.6	11.0	62%	95%	G3–4: 66%
Li et al. (20)	2022	R	41 Osimertinib 6 other *EGFR* TKIs 6 non-*EGFR* TKIs	53	1 Exon18 19 Exon19 2 Exon20 26 Exon21	21.4%	NA	13	NA	90%	NA
Xu Y et al. (21)	2021	R	Osimertinib 80mg/day	40	15 Exon19 23Exon21	40%	10	15.1	20%	95%	NA
Xu Z et al. (22)	2023	R	Furmonertinib 160mg/day	16	7 Exon19 6 Exon21	6.3%	4.3	NR	NA	NA	G3–4: 14.3%

LM, leptomeningeal metastasis; PFS, progression-free survival; OS, overall survival; R, retrospective; P, prospective; NSCLC, non-small cell lung cancer; m, months; EGFR, epidermal growth factor receptor; TKI, tyrosine kinase inhibitor; ORR, overall response rate; DCR, disease control rate; NR, not reached; NA, not applicable; G, grade of toxicity.

In the case described here, the patient received gefitinib adjuvant therapy for 15 months after pulmonary radical lobectomy, which was followed by intracranial progression only. Previous studies have revealed that the ability of gefitinib to penetrate the CNS is limited. Preclinical data show that gefitinib is distributed in the brain to a lesser extent than osimertinib (CSF/brain-to-blood ratio of exposure was 0.28 for ^[11C]^gefitinib and 2.62 for ^[11C]^osimertinib) (30). Several studies have reported that intracranial progression is likely to be associated with the poor ability of TKIs to diffuse through the BBB, resulting in pharmacokinetic failure (31). For the patient mentioned in this case, genetic testing of the surgical specimen in June 2020 revealed an *EGFR* exon 19 mutation. However, after intracranial progression, his plasma samples still tested positive for *EGFR*-activating mutations and negative for the T790M mutation. It has been reported that the frequency of T790M mutation is much lower in CSF lesions than in thoracic lesions (32). Therefore, we consider that the initial disease progression was probably due to the poor ability of gefitinib to penetrate the CNS, leading to the rapid development of intracranial lesions.

After the development of LM, the patient exhibited an ECOG PS of 3 with lethargy and a high intracranial burden. Given the poor prognosis of T790M-negative patients, systemic chemotherapy and ITC were not appropriate choices for the patient. In recent years, TKIs have shown extensive benefits for survival in patients with oncogenic driver mutations. Considering previous studies suggesting that a high dose of furmonertinib may be better in order to deliver a higher dosage to the CNS, we innovatively administered a high dose of furmonertinib to achieve better control of intracranial lesions and rapidly alleviate neurologic symptoms. After 5 days, the neurologic symptoms completely disappeared, and the patient regained self-care abilities. Eventually, the patient experienced an extended lifespan of 6 months, similar to that achieved with conventional therapy. This case implies that third-generation TKIs should be recommended for NSCLC patients with intracranial progression, especially patients with poorer physical status and limited tolerance for cytotoxic chemotherapy after the administration of first- or second-generation TKIs, regardless of T790M status. Given the dismal prognosis of NSCLC patients with LM, high-dose TKIs should be administered to achieve rapid disease control.

In the case described here, extracranial lesions exhibited no response to furmonertinib, while intracranial lesions were better controlled. The heterogeneity of resistance to TKIs in progressive disease is worth further exploration. The efficacy of furmonertinib on extracranial lesions can also be observed in patients treated with third-generation TKIs. Previous studies have indicated that heterogeneity of missense mutations between primary and metastatic lesions in NSCLC is frequent (33, 34). The ORR for extracranial lesions in patients with CNS metastases treated with furmonertinib has been found to be 77% (26), exceeding the ORRs of 56% in patients treated with osimertinib (27) and 61.5% in patients treated with aumolertinib (35). A case series in NSCLC has reported that ineffective TKI treatment could be explained in part by the discordance of molecular genetic profiles in lung adenocarcinoma with intrapulmonary metastases (36). We considered the possibility that extracranial resistance to furmonertinib could be attributed to the coexistence of the T790M mutation with other subclones. The *EGFR* C797S mutation is a common mutation site during progression under first- and second-generation *EGFR*-TKIs. A recent study has further indicated that, when the C797S mutation is in cis with the T790 mutation (i.e., on the same allele), the cancer cells are resistant to all *EGFR* TKIs, alone or in combination (37). In addition, several retrospective studies have observed a better clinical response and longer intracranial PFS in NSCLC and LM patients with T790M-positive CSF than in T790M-negative patients (15, 38). In this article, we have reported on observations of heterogeneity in resistance to third-generation TKIs between intracranial and extracranial lesions, and discussed the clinical implications. Current clinical trials in NSCLC with LM mostly exclude patients without the T790M mutation. For LM patients without acquired T790M mutation, it is important to closely monitor TKI resistance in clinical practice.

There are several limitations to this case study. The genetic status of the thoracic lesions could not be confirmed owing to the absence of re-biopsy of primary lesions, and the lack of CSF genotyping limited further interpretation of the heterogeneity of resistance to furmonertinib. Gene detection of CSF samples should be recommended owing to a perceived difference in genetic sequencing results between the plasma and CSF samples (32). Sequencing of CSF samples can improve diagnostic accuracy and therapeutic monitoring in LM. In this case, it was regrettable that we were not able to sequence the patient’s CSF. Because we did not reserve enough CSF for NGS, we would have needed a second lumbar puncture; the alternative option was to use peripheral blood as a replacement. Unfortunately, the patient refused a second lumbar puncture, and we had to sequence the peripheral blood.

## Conclusion

In conclusion, furmonertinib, a third-generation *EGFR* TKI with a strong ability to penetrate the BBB, may be effective in controlling LM and rapidly alleviating intracranial symptoms. However, further research is needed to determine the appropriate dosage and toxicity management strategies.

## Data availability statement

The original contributions presented in the study are included in the article/supplementary material. Further inquiries can be directed to the corresponding author.

## Ethics statement

Written informed consent was obtained from the individual(s) for the publication of any potentially identifiable images or data included in this article.

## Author contributions

TC was responsible for the conceptualization of the study, investigations, and writing of the original draft. JC was responsible for writing, reviewing, and editing. D-SL, Y-LS, H-JG, M-YF, and H-JG were responsible for performing the data analyses. K-JL and Y-MJ were responsible for visualization. All authors contributed to and approved the final version of the manuscript.
